# Supplementary material for: Allele frequency divergence reveals ubiquitous influence of positive selection in Drosophila
Source: PLoS Genet. 2021 Sep 30;17(9):e1009833. doi: 10.1371/journal.pgen.1009833 (PMC8509871; doi:10.1371/journal.pgen.1009833)
Supplement: S1 Text — This file contains supplemental text sections A-E. (PDF) [file pgen.1009833.s001.pdf]

Supplement to: Allele frequency divergence reveals ubiquitous influence of positive selection in *Drosophila*

Jason Bertram

September 24, 2021

A Frequency dependence of C for neutrally evolving alleles

Here we show that the allele frequency variance of neutrally evolving alleles has the form $\mathrm{Var}(\Delta_t p|p) = C_t p(1-p)$ where $\Delta_t p$ is the change in allele frequency over t generations, the variance is evaluated across loci with the same initial allele frequency p, and the variance coefficient C_t is independent of p. Neutrally evolving alleles are defined as having no effect on fitness and being in linkage equilibrium with alleles that do affect fitness.

Our argument is based on a generalization of the Cannings model [1]. Let y_i be the number of copies of an allele descended from the i'th copy of that allele t generations ago. There are a total of $Y = \sum_{i=1}^{X} y_i$ copies of the allele in the population, where X is the number of allele copies present t generations ago. The allele frequency change that occurs is $\Delta_t p = \frac{Y}{N(t)} - \frac{X}{N(0)}$, where $N(0)$ and $N(t)$ denote the census population size t generations ago and at the present time, respectively. To simplify our equations we assume a haploid population; for a diploid population we replace $N \to 2N$ without otherwise affecting our argument.

Since all loci share the same demography, there is no among-locus variance in $N(t)$. It follows that the among-locus variance in Y for alleles with the same initial frequency p (and hence same initial abundance X) can be written as $\mathrm{Var}(Y|p) = \mathrm{Cov}(Y, Y|p) = -\mathrm{Cov}(Y, N(t)-Y|p) = -\mathrm{Cov}(\sum_{i=1}^{X} y_i, \sum_{j=X+1}^{N} y_j|p) = -X(N-X)\overline{\mathrm{Cov}(y_i, y_j|p)}$, where $\overline{\mathrm{Cov}(y_i, y_j|p)} = \frac{1}{X(N-X)} \sum_{i=1}^{X} \sum_{X+1}^{N} \mathrm{Cov}(y_i, y_j|p)$ is the descendant number covariance averaged over allele copies. Consequently,

we have

$$
\begin{aligned}
\mathrm{Var}(\Delta p|p) &= \mathrm{Var}\left(Y/N(t) - X/N(0)|p\right) \\
&= \mathrm{Var}\left(Y/N(t)|p\right) \\
&= \frac{N(0)^2}{N(t)^2}\frac{\mathrm{Var}(Y|p)}{N(0)^2} \\
&= -\frac{N(0)^2}{N(t)^2}\overline{\mathrm{Cov}(y_i, y_j|p)}p(1-p). \qquad (1)
\end{aligned}
$$

To prove our claim that C_t is independent of p we therefore need to show that the averaged covariance $\overline{\mathrm{Cov}(y_i, y_j|p)}$ is independent of p.

Frequency-independent C_t is straightforward to show in the Cannings model [2]. Neutrality in the Cannings model takes the form of a symmetry assumption: the random variables y_i are assumed to be exchangeable (i.e. the joint probability distribution of the y_i is invariant with respect to rearrangement of their labels i; [3]). Exchangeability allows for a wide variety of marginal distributions for y_i (the allelic offspring distribution), giving the Cannings model enormous biological flexibility (e.g. [2]). When the y_i are exchangeable, $\overline{\mathrm{Cov}(y_i, y_j|p)} = \mathrm{Cov}(y_i, y_j|p)$ is independent of i, j for $i = 1, \ldots, X$ and $j = X+1, \ldots, N$ because $\mathrm{Var}(N(t)) = 0$ implies $\mathrm{Cov}(y_i, y_j|p) = -\sigma^2/(N(0)-1)$ where $\sigma^2 = \mathrm{Var}(y_1) = \ldots = \mathrm{Var}(y_{N(0)})$ is the variance in the number of allele descendants. This value of $\mathrm{Cov}(y_i, y_j|p)$ is independent of p because, from the definition of exhcangeability, which allele identities are attached to the y_i has no bearing on their joint probability distribution.

However, the Cannings exchangeability assumption has an important limitation as a model of genetic neutrality, namely that no non-neutral loci can be present in the genome. Non-neutral loci introduce fitness variation among genetic backgrounds which implies differences in the marginal distributions of the y_i corresponding to those genetic backgrounds. Nevertheless, provided that the loci over which $\mathrm{Var}(\Delta_t p|p)$ is being calculated are themselves neutral and in linkage equilibrium with loci under selection, $\overline{\mathrm{Cov}(y_i, y_j|p)}$ will still be frequency independent. To see this, note first that $\overline{\mathrm{Cov}(y_i, y_j|p)}$ is invariant with respect to reshuffling $i, j \to i', j'$ of the allele copy labels at each locus:

$$
\begin{aligned}
\overline{\mathrm{Cov}(y_i, y_j|p)} &= \frac{1}{X(N(0)-X)}\mathrm{Cov}(\sum_{i=1}^{X} y_i, \sum_{j=X+1}^{N} y_j) \\
&= \frac{1}{X(N(0)-X)}\mathrm{Cov}(\sum_{i'=1}^{X} y_{i'}, \sum_{j'=X+1}^{N} y_{j'}) \\
&= \overline{\mathrm{Cov}(y_{i'}, y_{j'}|p)} \qquad (2)
\end{aligned}
$$

In particular, at each locus we can assign i, j labels at random such that y_i and y_j are sampled at random from one focal and one non-focal background respectively. For neutral alleles in linkage equilibrium with non-neutral alleles, the focal and non-focal distributions of genetic backgrounds thus sampled are

identical. This leads to a situation analogous to strict exchangeability: the allele identities of the y_i have no bearing on the distribution of pairs y_i, y_j that enter the covariance implying that $\overline{\mathrm{Cov}(y_i, y_j|p)}$ is independent of p. This proves our claim.

B Population structure

We assume that the population under consideration consists of K subpopulations. Population allele frequencies can be written as a sum over subpopulations $p = \sum_{i=1}^{K} f_i p_i$, where the f_i are the proportional subpopulation abundances with $\sum_i f_i = 1$. Each subpopulation is assumed to evolve neutrally and independently such that $\mathrm{Var}(\Delta_t p_i | p_i = p_{i0}) = C_i(t) p_{i0}(1 - p_{i0})$ (A in S1 Text) where p_{i0} is the initial frequency in population i and $C_i(t)$ is independent of p_{i0}. The assumption of independent evolution implies no migration between subpopulations and hence maximal potential for subpopulation differentiation. We discuss the effects of migration further below.

The variance in population $\Delta_t p$ is evaluated for loci with a fixed initial population p, which means that the subpopulation p_{i0} may differ between loci. Using the law of total variance, we decompose the population variance into terms representing loci with different initial frequency vectors $\vec{p}_0 = (p_{00}, p_{10}, \ldots, p_{K0})$,

$$\mathrm{Var}(\Delta_t p | p) = E_{\vec{p}_0}[\mathrm{Var}(\Delta_t p | p, \vec{p}_0)] \tag{3}$$

since neutrality within each subpopulation implies $E[\Delta_t p | p, \vec{p}_0] = 0$. Evaluating the variance inside the expectation gives

$$\begin{aligned}
\mathrm{Var}(\Delta_t p | p, \vec{p}_0) &= \mathrm{Var}(\sum_{i=1}^{K} f_i \Delta_t p_i | p_{i0}) \\
&= \sum_{i=1}^{K} f_i^2 \mathrm{Var}(\Delta_t p_i | p_{i0}) \\
&= \sum_{i=1}^{K} f_i^2 C_i(t) p_{i0}(1 - p_{i0}) \qquad (4)
\end{aligned}$$

where in the first line we have used the independence of subpopulations (no covariances between the $\Delta_t p_i$).

Substituting this expression into (3) and using the fact that $E_{\vec{p}_0}[p_{i0}|p] = p$, we obtain

$$\begin{aligned}
\mathrm{Var}(\Delta_t p | p) &= \sum_{i=1}^{K} f_i^2 C_i(t) \left(p - E_{\vec{p}_0}[p_{i0}^2 | p]\right) \\
&= \sum_{i=1}^{K} f_i^2 C_i(t) \left(p(1-p) - \mathrm{Var}_{\vec{p}_0}[p_{i0}|p]\right) \qquad (5)
\end{aligned}$$

Deviations from binomial variance due to population structure thus arise due to the subpopulation variances $\mathrm{Var}_{\vec{p}_0}[p_{i0}|p]$, i.e. the among-locus variance in the subpopulation allele frequencies among alleles with population frequency p at $t = 0$. In general these variances depend on the structure of genetic variation across subpopulations. To gain some intuition, suppose that the subpopulation-specific variance coefficients $C_i(t)$ are inversely proportional to census population size such that $f_i C_i(t) = A$ has the same value in each subpopulation. We then obtain

$$\begin{aligned}
\mathrm{Var}(\Delta_t p|p) &= A E_{\vec{p}_0|p}\left[\sum_{i=1}^{K} f_i p_{i0}(1-p_{i0})\right] \\
&= Ap(1-p)\left(1 - E_{\vec{p}_0}[G_{ST}|p]\right) \qquad (6)
\end{aligned}$$

where $G_{ST} = 1 - \sum_{i=1}^{K} f_i p_{i0}(1-p_{i0})/[p(1-p)]$ is Nei's definition of the fixation index at one locus [4]. In the case of a single biallelic locus, G_{ST} can span almost the entire $[0, 1]$ range at intermediate population frequencies p, but approaches zero as $p \to 1$ [5]. Since the G_{ST} term is subtracted in the above equation, this suggests that population structure tends to create a variance deficit at intermediate frequencies compared to a binomial (the opposite of the variance excess described in the main text).

We now drop the $f_i C_i(t) = A$ restriction used above to connect our analysis to G_{ST}, and argue that $\mathrm{Var}_{\vec{p}_0}[p_{i0}|p]$ grows faster than binomial as $p \to \frac{1}{2}$ under broadly applicable biological conditions.

We first assume that the allele frequencies in each subpopulation are independent. Since new mutations arise at low frequency and most are rapidly lost before reaching intermediate frequencies, the distribution of allele frequencies (the folded site frequency spectrum) in each subpopulation is strictly decreasing with increasing p. The joint distribution of p_{i0}, and the frequency of the same allele in the rest of the population $\hat{p}_{i0} = \sum_{j \neq i} f_j p_{j0}$, is thus the product of two distributions, $P(p_{i0})$ and $\hat{P}(\hat{p}_{i0})$, each strictly decreasing with increasing p. The variance $\mathrm{Var}_{\vec{p}_0}[p_{i0}|p]$ is determined by the distribution of p_{i0} along the line $f_i p_{i0} + \hat{p}_{i0} = p$ i.e. $P(p_{i0}|p) = P(p_{i0}, \hat{p}_{i0} = p - f_i p_{i0}) = P(p_{i0})\hat{P}(\hat{p}_{i0} = p - f_i p_{i0})$ where $0 \leq p_{i0} \leq \min\{p/f_i, 1\}$ and $f_i \leq \frac{1}{2}$. It follows that $\frac{dP(p_{i0}|p)}{dp_{i0}} = P'(p_{i0}) - f_i \hat{P}'(p - f_i p_{i0})$, where the derivatives P' and $\hat{P}'$ are negative. The distribution $P(p_{i0}|p)$ must therefore have a mode at $p_{i0} = 0$ or $p_{i0} = \min\{p/f_i, 1\}$ or both, but nowhere else. The variance $\mathrm{Var}_{\vec{p}_0}[p_{i0}|p]$ will be greatest in the two-mode case, so we assume a single mode at $p_{i0} = 0$ without loss of generality i.e. strictly decreasing $P(p_{i0}|p)$ with increasing p_{i0}. Then, as p increases $\mathrm{Var}_{\vec{p}_0}[p_{i0}|p]$ grows at rate approximately proportional to p^2, because the most probable values lie near $p_{i0} = 0$ with an associated square deviation of $(p_{i0} - p)^2 = p^2$. Thus, when subpopulatoin allele frequencies are independent, $\mathrm{Var}_{\vec{p}_0}[p_{i0}|p]$ increases much faster than binomial $\sim p(1-p)$ as $p \to \frac{1}{2}$, in accord with what we would expect from the frequency dependence of G_{ST} [5].

Allele frequency correlations among subpopulations can be caused by shared history or migration. In the case where isolated subpopulations descend from a

shared ancestral population, allele frequencies will initially diverge binomially, tending to the independent case above as correlations decay due to drift.

The effects of migration among subpopulations are more complex, since subpopulations no longer evolve independently, and there can be nontrivial migratory relationships between subpopulations due e.g. to spatial structure. Given this complexity and our primary focus on closed laboratory populations, we leave detailed analysis of this scenario for future work. We note, however, that migration acts to eliminate subpopulation differentiation, moving the evolutionary dynamics in the population closer to admixture. It would therefore be surprising if migration between subpopulations created non-binomial allele frequency divergence at the population level.

C Fluctuating selection and temporal autocovariances

In this section we give further details of the relative contributions of persistent directional selection versus fluctuating selection to the selective divergence over $t > 1$ generations.

We assume that the total selection coefficients s_i are random variables with probability distribution that may depend on time i and locus l. The expectation of this distribution, denoted $E[s_i|l]$, represents the consistent selective pressure in generation i at locus l. This consistent pressure could be constant over time, cycle periodically, or have no temporal pattern. The deviation of s_i from $E[s_i|l]$, denoted $\epsilon_i = s_i - E[s_i|l]$, represents a random selective component arising from unpredictable environmental factors or randomness in the genetic backgrounds experienced by each allele due to genetic drift, assortment and recombination. The variance of ϵ_i, $\mathrm{Var}(\epsilon_i|l)$, measures the intensity of stochastic fluctuations in s_i at locus l. Both the time dependence of the expected total selection coefficients $E[s_i|l]$ and the stochastic fluctuations represented by ϵ_i are forms of fluctuating selection.

Suppose that $s_0, \ldots, s_{t-1}$ are independent and identically distributed (iid) random variables (allowed to be different between loci). This represents a situation where there is no systematic time-dependence in the s_i; the only time dependence is due to temporally-uncorrelated fluctuations. The within-generation contribution to divergence is then given by

$$\begin{aligned}\sum_{i=0}^{t-1} \mathrm{Var}(s_i) &= t\mathrm{Var}(s_0) \\ &= t\left[E_l(\mathrm{Var}[\epsilon_0|l]) + \mathrm{Var}_l(E[s_0|l])\right]. \qquad (7)\end{aligned}$$

which grows linearly with time analogous to a random walk. This is to be expected since the within-generation divergence represents the effects of among-locus fitness variation solely on a generation-by-generation basis regardless of how that variation is correlated over time. Note that even in the case where

there is no systematic among-locus variation $\mathrm{Var}_l(E[s_0|l]) = 0$, there remains a divergence due to selective fluctuations with a non-binomial frequency dependence.

On the other hand, the between-generation contribution to selective divergence in the iid case is

$$\begin{aligned}\sum_{i\neq j}\mathrm{Cov}(s_i, s_j) &= \sum_{i\neq j}\mathrm{Cov}(E[s_i|l], E[s_j|l]) \\ &= t(t-1)\mathrm{Cov}(E[s_i|l], E[s_j|l]) \\ &= t(t-1)\mathrm{Var}_l(E[s_0|l]). \qquad (8)\end{aligned}$$

Sustained directional selection thus generates divergence an order of magnitude faster than fluctuating selection (growing quadratically over time). Therefore, the overall divergence starts to be dominated by between-generation covariances in the iid case when t grows large enough that $t^2 \gg t$. The empirical studies in the main text have $t \sim 10$, which is large enough that the covariance contribution would dominate for iid s_i.

D Selective perturbation of the drift variance

Here we evaluate the influence of selection on the drift contribution to allele frequency divergence, $E_s[\mathrm{Var}(\Delta_t p|p, \{s_0, \dots, s_{t-1}\})]$. We do so by deriving a recursion for $\mathrm{Var}(\Delta_t p|p, \{s_0, \dots, s_{t-1}\}) = \mathrm{Var}(p_t|p_0, \{s_0, \dots, s_{t-1}\})$ going backwards along the allele frequency trajectory $p_t, \dots, p_1, p_0$ where $p_0 = p$.

We start with the neutral case $s_0 = \cdots = s_{t-1} = 0$. Applying the law of total variance to account for the variance added to p_t due to the allele frequency variance from the preceding generation p_{t-1}, we have

$$\begin{aligned}\mathrm{Var}(p_t|p_0) &= E_{p_{t-1}}[\mathrm{Var}(p_t|p_{t-1}, p_0)] + \mathrm{Var}_{p_{t-1}}(E[p_t|p_{t-1}, p_0]) \\ &= E[d_{t-1}p_{t-1}(1-p_{t-1})|p_0] + \mathrm{Var}(p_{t-1}|p_0) \\ &= d_{t-1}(p_0 - p_0^2 - \mathrm{Var}(p_{t-1}|p_0)) + \mathrm{Var}(p_{t-1}|p_0) \\ &= d_{t-1}p_0(1-p_0) + (1-d_{t-1})\mathrm{Var}(p_{t-1}|p_0) \\ &= p_0(1-p_0)\sum_{i=0}^{t-1} d_i \prod_{j=i+1}^{t-1}(1-d_j) \qquad (9)\end{aligned}$$

where d_{t-1} is the variance coefficient for $\mathrm{Var}(p_t|p_{t-1})$ and the last line is obtained by recursion. The variance coefficient for $\mathrm{Var}(p_t|p_0)$ is thus $D_t = \sum_{i=0}^{t-1} d_i \prod_{j=i+1}^{t-1}(1-d_j)$.

We now evaluate how selection changes Eq. (9). We only evaluate the first order effects of selection since we focus on the scenario where selective allele frequency changes are small over the interval t (i.e. $\sum_{i=0}^{t-1} s_i \ll 1$ and $|s_i| \ll 1$).

First, selection perturbs the variance over one generation such that $\mathrm{Var}(p_i|p_{i-1}, p_0) = d_{i-1}(p_{i-1}(1-p_{i-1}) + \delta_{i-1})$ where the perturbation δ_{i-1} is of order s_{i-1} (for brevity we do not explicitly write the conditional dependence on $\{s_0, \dots, s_{t-1}\}$).

Second, we now have (to first order in s)

$$
\begin{aligned}
\mathrm{Var}_{p_{i-1}}(E[p_i|p_{i-1}, p_0]) &= \mathrm{Var}_{p_{i-1}}(p_{i-1} + s_{i-1}p_{i-1}(1-p_{i-1})) \\
&\approx \mathrm{Var}_{p_{i-1}}(p_{i-1}) + 2s_{i-1}\mathrm{Cov}(p_{i-1}, p_{i-1}(1-p_{i-1})) \\
&= \mathrm{Var}_{p_{i-1}}(p_{i-1}) + 2s_{i-1}[\mathrm{Var}(p_{i-1}) - \mathrm{Cov}(p_{i-1}, p_{i-1}^2)].
\end{aligned}
\tag{10}
$$

The remaining covariance can be written as

$$
\mathrm{Cov}(p_{i-1}, p_{i-1}^2) = E[(p_{i-1} - p_{i-1})^3] + 2E[p_{i-1}]\mathrm{Var}(p_{i-1}) \tag{11}
$$

We assume that the initial cohort allele frequency p_0 is sufficiently far from fixation, and the duration of divergence t is small enough, that p_i is close to symmetrically distributed for all $1 \leq i \leq t-1$, such that the skewness term $E[(p_{i-1} - p_{i-1})^3]$ can be neglected. Moreover, the selective change to $E[p_{i-1}]$ is $O(s^2)$ since the covariance is multiplied by s_{i-1}. We thus obtain,

$$
\mathrm{Var}_{p_{t-1}}(E[p_t|p_{t-1}, p_0,]) \approx (1 + 2s_{t-1}(1-2p_0))\mathrm{Var}(p_{t-1}) \tag{12}
$$

to first order in s. Consequently, the neutral result Eq. (9) becomes

$$
\begin{aligned}
\mathrm{Var}(p_t|p_0) &\approx \sum_{i=0}^{t-1} d_i\alpha_i \prod_{j=i+1}^{t-1}(1 - d_j + 2s_j(1-2p_0)) \\
&\approx \sum_{i=0}^{t-1} d_i\alpha_i \prod_{j=i+1}^{t-1}(1-d_j)\left[1 + (1-2p_0)\sum_{j=i+1}^{t-1}\frac{2s_j}{1-d_j}\right]
\end{aligned}
\tag{13}
$$

where $\alpha_i = p_i(1-p_i) + \delta_i$.

Taking the expectation E_s and substituting $E[p_i] \approx p_0 + p_0(1-p_0)\sum_{j=0}^{i-1} s_j$, which implies $E[\alpha_i] \approx p_0(1-p_0)[1 + (1-2p_0)\sum_{j=0}^{i-1} s_j] + E[\delta_i]$, we obtain

$$
\begin{aligned}
E_s[\mathrm{Var}(p_t|p_0)] \approx p(1-p_0)\sum_{i=0}^{t-1} d_i \prod_{j=i+1}^{t-1}(1-d_j)\times \\
\left[1 + (1-2p_0)\left(\sum_{j=0}^{i-1}E[s_j] + \frac{E[\delta_i]}{p_0(1-p_0)(1-2p_0)} + 2\sum_{j=i+1}^{t-1}\frac{E[s_j]}{1-d_j}\right)\right]
\end{aligned}
\tag{14}
$$

to first order in s.

Observe that the term in large parentheses in Eq. (14) is closely related to the expected selection coefficient for the accumulated allele frequency change after t generations, $E[\sum_{i=0}^{t-1} s_i]$. One complication is the presence of the within-generation perturbation δ_i, the exact form of which will in general depend on population dynamic specifics. However, the contribution from the term containing δ_i will not be important when $t \gg 1$, as is the case in the present study

($t \sim 10$ generations). Moreover, note that in the Wright-Fisher model, selection is usually incorporated by applying the selective perturbation to gamete frequencies prior to binomial sampling; this gives $\mathrm{Var}(p_{i+1}|p_i, p_0) = d_i p_{i+1}(1 - p_{i+1})$ and thus $\delta_i = p_i(1 - p_i)(1 - 2p_i)s_i$; hence $E[\delta_i]/[p_0(1 - p_0)(1 - 2p_0)] \approx s_i$. Similar behavior occurs in the continuous-time Moran model (see Appendix in [6]). Thus, the δ_i term could plausibly be proportional to s_i (with no frequency dependence) in many populations.

Another complication is the extra weight given to later generations due to the doubling factor in the term $2\sum_{j=i+1}^{t-1} \frac{E[s_j]}{1-d_j} \approx 2\sum_{j=i+1}^{t-1} E[s_j]$. This weighting means that the term in large parentheses in Eq. (14) will have a value close to $E[\sum_{i=0}^{t-1} s_i]$. That is,

$$E_s[\mathrm{Var}(p_t|p_0)] \approx D_t p(1 - p_0)\left(1 + c(1 - 2p_0)E[\sum_{i=0}^{t-1} s_i]\right) \tag{15}$$

where c is a constant factor of order 1.

E Measurement error

In this section we show that the major sources of allele frequency measurement error do not systematically alter the frequency independence of C_t in main text Eq. (1). Population allele frequencies are denoted p while estimated allele frequencies are denoted $\hat{p}$.

Suppose that the measurement variance satisfies $\mathrm{Var}(\hat{p}|p) = Mp(1-p)$ where M is frequency independent and can also differ between generations. Applying the law of total variance (for any two random variables X and Y, $\mathrm{Var}(Y) = E_X[\mathrm{Var}_Y(Y|X)] + \mathrm{Var}_Y(E_X[Y|X])$), alleles with the same initial frequency p_0 will have variance,

$$\begin{aligned}
\mathrm{Var}(\Delta_t\hat{p}|p_0) &= \mathrm{Var}(\hat{p}_t - \hat{p}_0|p_0) \\
&= \mathrm{Var}(\hat{p}_t|p_0) + \mathrm{Var}(\hat{p}_0|p_0) \\
&= E_{p_t}[\mathrm{Var}(\hat{p}_t|p_t, p_0)] + \mathrm{Var}_{p_t}(E[\hat{p}_t|p_t, p_0]) + M_0 p_0(1 - p_0) \\
&= E_{p_t}[M_t p_t(1 - p_t)|p_0] + \mathrm{Var}_{p_t}(p_t|p_0) + M_0 p_0(1 - p_0) \\
&= M_t(E[p_t|p_0] - E[p_t^2|p_0]) + C_t p_0(1 - p_0) + M_0 p_0(1 - p_0) \\
&= M_t(E[p_t|p_0] - (\mathrm{Var}(p_t|p_0) + E[p_t|p_0]^2)) + (C_t + M_0)p_0(1 - p_0) \\
&= (C_t + M_0 + M_t - M_t C_t)\, p_0(1 - p_0) \qquad (16)
\end{aligned}$$

where we have used the fact that $E[p_t|p_0] = p_0$ for neutral alleles. Therefore, measurement error does not change the frequency dependence of neutral cohort variances, provided that our assumption of frequency independent M_t is true. We now show the latter accounting for different sources of error. Following [7], we divide measurement error into three main sources that will be considered in turn: population sampling, pooling and amplification of the sampled DNA, and finite read depth.

Population sampling

Random sampling of individuals generates a binomial sampling error, such that the sample allele frequency p_S has variance $\mathrm{Var}(p_S|p) = p(1-p)/n_S$ where n_S is the sample size. Using the law of total variance, the contribution of this sampling error is

$$\begin{aligned}\mathrm{Var}(\hat{p}|p) &= E_{p_S}[\mathrm{Var}(\hat{p}|p_S)] + \mathrm{Var}_{p_S}[E(\hat{p}|p_S)] \\ &= E_{p_S}[\mathrm{Var}(\hat{p}|p_S)] + p(1-p)/n_s \qquad (17)\end{aligned}$$

The term $\mathrm{Var}(\hat{p}|p_S)$ represents the error in $\hat{p}$ introduced after population sampling, which we now evaluate.

Read sampling

Let r be the number of reads of the reference allele A recorded by the sequencer at a locus with a total of r_T reads. The allele frequency estimate of A is $\hat{p} = r/r_T$. We initially restrict our attention to loci that have the same read depth r_T; below we incorporate the effects of among-locus variation in r_T. Reads are assumed to be generated by random sampling with replacement from the DNA pool. Reference allele reads are thus binomially distributed $r \sim \mathrm{Bin}(r_T, p_P)$ where p_P is the allele frequency of A in the sequenced DNA pool.

If all allele copies sampled from the population contribute equally to the sequenced DNA pool, then $p_P = p_S$. Errors introduced during pooling and amplification cause unequal contributions and thus variance in p_P among sequenced loci. We assume that pooling+amplification error do not systematically bias allele frequencies such that $E_{p_P}[p_P] = p_S$. Applying the law total variance to $\hat{p}$ for fixed r_T gives

$$\begin{aligned}\mathrm{Var}(\hat{p}|p_S, r_T) &= \frac{\mathrm{Var}(r|p_S, r_T)}{r_T^2} \\ &= \frac{E_{p_P}[\mathrm{Var}_r(r|p_S, p_P, r_T)] + \mathrm{Var}_{p_P}(E_r[r|p_S, p_P, r_T])}{r_T^2} \\ &= \frac{E_{p_P}[r_T p_P(1-p_P)] + \mathrm{Var}_{p_P}(r_T p_P|p_S)}{r_T^2} \\ &= \frac{p_S(1-p_S) + (r_T - 1)\mathrm{Var}(p_P|p_S)}{r_T} \qquad (18)\end{aligned}$$

where we have used the identity $E[X^2] = \mathrm{Var}[X] + E[X]^2$ in the last line. Note that the conditionality on r_T has been dropped in $\mathrm{Var}_{p_P}(p_P|p_S)$ because the DNA pool frequency p_P is independent of r_T. We now incorporate the effects of pooling and amplification to show that $\mathrm{Var}_{p_P}(p_P|p_S) \propto p_S(1-p_S)$.

Pooling+amplification

Let F_i denote the proportion of the DNA pool at the focal locus originating from the i'th sampled allele copy, where $i = 1, \ldots, 2n_S$ for diploids. In the

case of equal contributions from each sampled allele copy we simply have $F_i = 1/2n_S$, but in general the F_i are random variables with unknown probability distributions denoted $P(F_i)$ (ref. [7] assumes a Dirichlet distribution for the F_i). We have $p_P = \sum_{i=1}^{2n_S p_S} F_i$ where $2n_S p_S$ is the number of sampled allele copies containing the focal allele (i.e. we order the haplotype labels i such that the reference allele copies appear first). Since the F_i sum to one, we have analogous to (1),

$$\text{Var}_{p_P}(p_P|p_S) = -\overline{\text{Cov}(F_i, F_j)}p_S(1-p_S) \tag{19}$$

where $\overline{\text{Cov}(F_i, F_j)} = \frac{1}{4n_S^2 p_S(1-p_S)} \sum_i^{2n_S p_S} \sum_j^{2n_S(1-p_S)} \text{Cov}(F_i, F_j)$ is the mean covariance of the DNA pool contributions between the focal and non-focal alleles.

Similar to exchangeability in the Cannings model, pooling and amplification are blind to allele copy identity and should therefore affect allele copy probabilities symmetrically (see A in S1 Text). This implies $\overline{\text{Cov}(x_i, x_j)} = \text{Cov}(x_i, x_j) = \text{Var}(F_i)/(2n_s - 1)$ independent of i and p_S. Combining the this result with (18) we therefore obtain

$$\text{Var}(\hat{p}|p_S, r_T) = f(r_T)p_S(1-p_S) \tag{20}$$

where $f(r_T)$ is independent of p_S.

Variable read depth

Read depth varies across loci according to some probability distribution $P(r_T)$. This variability in incorporated by integrating out the dependence on r_T

$$\text{Var}(\hat{p}|p_S) = \int \text{Var}(\hat{p}|p_S, r_T)P(r_T)dr_T = f p_S(1-p_S) \tag{21}$$

where $f = \int f(r_T)dr_T$. Substituting this result into (17) we finally obtain

$$\text{Var}(\hat{p}|p) = \left(\frac{1}{n_S} + f + \frac{f}{n_S}\right)p(1-p), \tag{22}$$

where the term multiplying $p(1-p)$ is frequency-independent as claimed at the start of S2.

References

1. Cannings C. The latent roots of certain Markov chains arising in genetics: a new approach, I. Haploid models. Advances in Applied Probability. 1974;6(2):260–290.

2. Der R, Epstein CL, Plotkin JB. Generalized population models and the nature of genetic drift. Theoretical population biology. 2011;80(2):80–99.

3. Chow YS, Teicher H. Probability theory: independence, interchangeability, martingales. Springer Science & Business Media; 2012.

4. Nei M. Analysis of gene diversity in subdivided populations. Proceedings of the National Academy of Sciences. 1973;70(12):3321–3323.

5. Alcala N, Rosenberg NA. Mathematical Constraints on FST: Biallelic Markers in Arbitrarily Many Populations. Genetics. 2017;206(3):1581–1600. doi:10.1534/genetics.116.199141.

6. Feder AF, Kryazhimskiy S, Plotkin JB. Identifying signatures of selection in genetic time series. Genetics. 2014;196(2):509–522.

7. Gautier M, Foucaud J, Gharbi K, Cézard T, Galan M, Loiseau A, et al. Estimation of population allele frequencies from next-generation sequencing data: pool-versus individual-based genotyping. Molecular Ecology. 2013;22(14):3766–3779.
