# Supplementary figures and images for: Allele frequency divergence reveals ubiquitous influence of positive selection in Drosophila
Source: PLoS Genet. 2021 Sep 30;17(9):e1009833. doi: 10.1371/journal.pgen.1009833 (PMC8509871; doi:10.1371/journal.pgen.1009833)

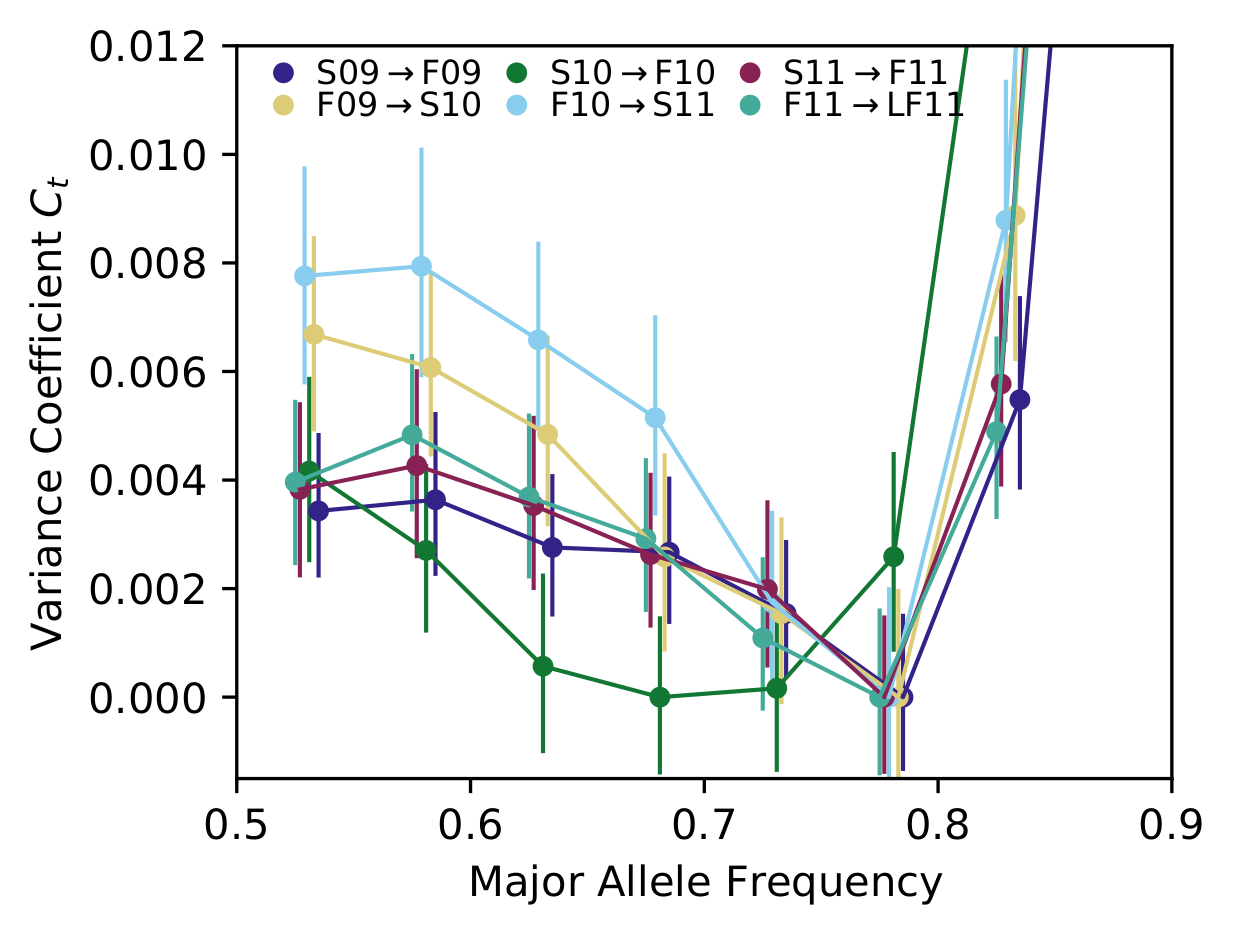

Supplement: S1 Fig — Same as Fig 3 but for the Bergland et al. data. Each curve represents a different seasonal iterate e.g. summer 2009 to fall 2009. (TIF) [file pgen.1009833.s002.tif]

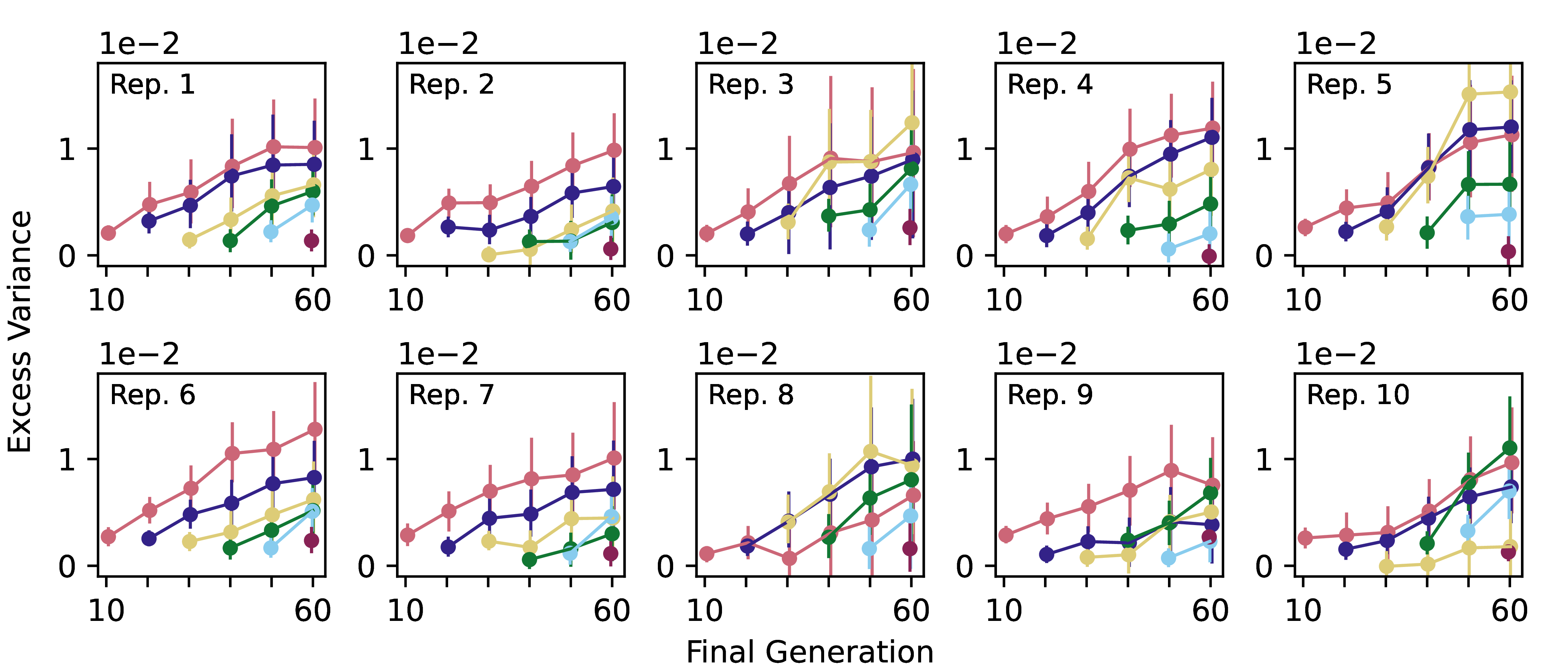

Supplement: S2 Fig — Same as Fig 4A but including all 10 replicates from Barghi et al. (TIF) [file pgen.1009833.s003.tif]
