# Supplementary material for: Allele frequency divergence reveals ubiquitous influence of positive selection in Drosophila
Source: PLoS Genet. 2021 Sep 30;17(9):e1009833. doi: 10.1371/journal.pgen.1009833 (PMC8509871; doi:10.1371/journal.pgen.1009833)
Supplement: Attachment — Submitted filename: response_to_reviews.pdf [file pgen.1009833.s004.pdf]

To the Editor,

We thank you and the two reviewers for your constructive feedback. We agree with all points raised and have revised the manuscript accordingly. Both major structural concerns have been addressed (migrating the fluctuating selection material from the supplement, moving simulations ahead of data). We have also created a new conceptual figure that summarizes the essential idea of our approach. We believe that these changes have substantially improved the accessibility of the manuscript.

Below we include a full response (italics) to the reviews.

Best Wishes,

Jason Bertram

Dear Dr Bertram,

Thank you very much for submitting your Research Article entitled 'Allele frequency divergence reveals ubiquitous influence of positive selection in Drosophila' to PLOS Genetics.

The manuscript was fully evaluated at the editorial level and by independent peer reviewers. The reviewers appreciated the attention to an important problem, but raised some substantial concerns about the current manuscript. Based on the reviews, we will not be able to accept this version of the manuscript, but we would be willing to review a much-revised version. We cannot, of course, promise publication at that time.

Yours sincerely,

Alex Buerkle
Associate Editor
PLOS Genetics

Bret Payseur
Section Editor: Evolution
PLOS Genetics

This manuscript has been reviewed by two referees, both of whom find the theoretical, methodological, and empirical results to be interesting. The reviews offer a number of suggestions for improvement of the manuscript, including encouragement to move material from the Supplement/Appendix into the main paper and to develop one or more visualizations to communicate the core result. They also raise some questions that are important for clarity and evaluation of the manuscript.

Reviewer #1: Bertram develops a theoretical partitioning of allele frequency change to distinguish genetic drift from natural selection from time series data, which here is SNP allele frequencies scored over time within an evolving population. The signal is that selection elevates the magnitude of change at intermediate allele frequencies relative to drift. He applies the approach to allele frequency measurements from laboratory and wild Drosophila populations and finds that selection has genome-wide effects. This paper is worthy of publication because it illustrates an important way that linked selection / genetic draft cannot be subsumed into an "effective population size effect." The applications to Drosophila demonstrate the genomic extent of this process. I have two major suggestions and several minor comments.

I. The paper has the structure of a short-format paper (e.g. Nature or PNAS). Plos Genetics allows a more rigorous treatment of a topic within the main body of the paper. For this reason, I think Appendix S3 should be moved into the main paper. The equations of S3 provide the reader with a far more explicit understanding of the parts of eq 4. It is important to see the sources of variability (and covariation) in selection coefficients, both among loci and through time, as determinants of outcomes. It also sets up an important contrast for the application to Drosophila data. Temporally fluctuating selection (of a specific flavor) is implicated for the Bergland et al field studies but not for the laboratory Evolve-and-Resequence studies. These differences are discussed (e.g. lines 207 onward, lines 307-325) but could be explained more clearly by reference to previous in-text equations.

We have migrated the bulk of S3 to the results section as suggested (a few technical details remain in S3). We agree that this is considerably improves the exposition of constant versus fluctuating selection. The later sections now refer directly to the results section as suggested.

II. The semantic classifications of selection are inconsistent and confusing. For example, Lines 12-14 set up selection as positive or negative. Directional selection is always positive on one allele and negative on the other. Here, the idea seems to involve the initial allele frequency of the favored allele. If so, that should be defined explicitly.

We take negative selection (as a description of a selective regime rather than the sign of selection coefficients on individual alleles) to mean the continual removal of unconditionally mutations. We have significantly expanded our discussion of these points, particularly in the new simulation section. Throughout we have revised the wording to be clearer about our meaning.

At other points in the manuscript, the author contrasts positive selection to "purifying selection." The latter term is typically associated with mutation-selection balance. Purifying selection is where selection acts to eliminate variation. Its usually considered as the alternative to balancing selection (where selection acts to maintain polymorphism), but that may be the meaning here.

Due to these connotations, we have removed the term "purifying selection" from the manuscript.

Perhaps for terminology reasons, I also struggled to follow the section of Lines 253-272. The point seems to be that "selection acting continuously to purge deleterious alleles" does not explain the results. Are the hypothesized mutations unconditionally deleterious?

Correct. See preceding comment.

If so, they would never have made it into the studies in the first place. Mutations rare at time zero and then stay rare would be filtered during variant calling and thus never make it into the analyzed datasets.

Perhaps, but note that the patterns we document are dominated by the behavior of neutral mutations. Selected mutations do not need to be included in our dataset for us to see the effects they are having on neutral mutations.

If the mutations were at intermediate frequency at the initiation of the study (time = 0), they would not likely have been unconditionally deleterious. One allele may have become deleterious in the lab environment in which the population was maintained if it was different (or even just more constant) than the ancestral environment. However, I am not sure how this scenario is distinct from “positive selection.”

We would agree that this is a form of positive selection in the sense we have used it. That is, it is not simply the routine purging of an influx of deleterious mutations. We cannot think of a simple term that would capture these subtleties so we have instead taken the approach of being more explicit about our meaning (see above).

Other comments:

Lines 27-29: “Numerous methods exist for inferring selection coefficients from allele frequency time series [10, 17–24], but are only reliable for selection that is strong relative to the intensity of random, non-selective allele frequency change (random genetic drift).”
For samples from natural populations (millions of individuals), the noise from finite sampling (limited numbers of individuals taken plus limited sequencing coverage) is likely to be more important problem than genetic drift in terms of the power of outlier tests.

We agree and have added this point to the sentence.

Line 370: Does “eliminate” mean ignore here? I cannot tell if this is a mathematical operation or the term has simply been dropped.

The term has been dropped. We have replaced “eliminate” with “drop”.

Reviewer #2: This MS develops a framework for testing temporal fluctuations in allele frequency due to selection. This framework uses the the variance in allele frequencies, conditional on starting frequency and shows using theory, simulation, and real data that strong temporally variable selection occurs over short time periods. Overall the MS is well written and I think that the results are relevant to a broad audience.

I think that the MS could be restructured a bit to make it more approachable to the non-theoretician. I still struggle to understand why the excess variance is frequency dependent. Can the author put together a conceptual figure that will help motivate an intuitive understanding of the results? I think that this would be crucial for this paper to be accessible to the PloS G readership.

We agree and have added a new figure (now Figure 1) in the revised manuscript which summarizes the essential ideas of our approach.

I also think that the paper could be re-ordered. First theory, then simulations, then data. Having the simulations at the end feels like an afterthought. However for people who might struggle with the maths, the simulation results very striking and might help the reader trust the importance of the results better.

We agree and have placed simulations ahead of data.

Minor comments
Line 143: "that is not important to our results". Do you mean "that is important to our results"? If you mean what you wrote, why should I care to read something that is not important?

We agree this text was confusing. It has been removed.

Line 185-186: "Similar results are found in a wild D. melanogaster population (not shown in Fig 1)." It is a little funny to read this sentence and then not see the results. Why didn't you include this dataset in Fig 1?

We have included a supplemental figure for curious readers (Fig. S1). Elevated variance in this data can be seen directly in figure 4 as well. We decided to leave out this data from Figure 1 (now figure 3) because it is of quite a different form: there no replicates at each time step, so we would need to show different timesteps. This is a lot for the reader to parse and is distracting from our message at this point.

Fig 4. What the 1e-2, 1e-4, and stars refer to?

The 1e-2 and 1e-4 show the overall scale of the axis (used throughout the manuscript). We think this was only confusing here because of the stars. We have added this text to the Fig. 4 caption to clarify: "Stars indicate which panel A simulations are shown in panels B and C respectively."
